# 

**Published:** 1891-11

**Authors:** 


					﻿Society Notes.
The Northwest Texas Medical Association will hold its
regular semi-annual meeting at Cisco, on the first Tuesday in
December, convening at 3 p. m. The following papers will be
read and discussed:
Pneumonia—Dr. Vance; discussion opened by Dr. Florence.
Puerperal Fever—Dr. Harrington; discussion opened by Dr.
Isbell.
Fracture of Thigh—Dr. W. M. Powell; discussion opened by
Dr. Rodman.
Placenta Praevia—Dr. Salmon; discussion opened by Dr. Stout.
New Therapeutical Remedies—Dr. R. G. Powell; discussion
opened by Dr. Pipkin.
If not already a member, attend this meeting, and we guar-
antee you will be amply repaid for your time.
P. C. Coleman, Secretary.
C. F. Paine, President.
Seventeenth Quarterly Meeting of the Austin District
Medical Society, and joint session with the Central Texas Medi-
cal Association, at Austin, Texas, Thursday, December 17, 1891:
Dear Doctor:—It affords us pleasure to announce that the
Central Texas Medical Association will meet with us in joint
session at this, our seventeenth quarterly meeting, and partici-
pate in the programme here subjoined. The Central Texas As-
sociation meets quarterly, at Waco, and is one of the largest and
most influential medical organizations in the State, and numbers
amongst its members some of the ablest men in the profession.
It need hardly be said that an unusually interesting and profit-
able time is anticipated. We will have a large attendance, and
you are cordially invited to be present; and if you are not al-
ready a member, join us, and take part in the proceedings.
You will observe that this is our annual meeting, at which
officers for the ensuing year are to be elected, and the usual
banquet given, Bear in mind, also, that the payment of annual
dues will be in order.
Married.—At Dallas, November 4th inst., at the residence of
the bride’s parents, Dr. and Mrs. E. D- Thompson, Dr. W. A.
Howard, of Waco, to Miss Fannie Thompson, of Dallas.
Died.—At his hotne near Willis Point, Texas, Sunday, No-
vember ist, inst., after a brief illness, Dr. W. T. Strain.
Dr. Strain was a young man, not over thirty years of age, and
a man of rare promise, as well as of unusual ability. His death
was as unexpected as it was deplored. He had suffered a fall
some time previously, but it was thought he had sustained no
injury. In two weeks from date of fall, he had a chill, followed
by another next day, and died at 2.30 p. m. Sunday, November
ist, it is supposed, of blood poisoning. Dr. Strain was a mem-
ber of the Texas State Medical Association, and of the Terrell
Medical Society.
I certify that I print, bind and deliver Daniel’s Texas Med-
ical Journal as follows: September issue, one thousand and
fourteen copies. Octobei issue, one thousand and nine copies.
November issue, one thousand and six copies, making in the
three editions, three thousand and twenty-nine copies (3029.)
Eugene Von Boeckmann.
Austin, Texas, Nov. 75, 1891.
Sworn to and subscribed before me. this 25th day of Novem-
ber, A. D. 1891.	Henry E. Shelley.
[seal.]	Notary Public, Travis Co., Texas.
				

